# Ubiquitin-Conjugating Enzyme E2 E Inhibits the Accumulation of Rice Stripe Virus in *Laodelphax striatellus* (Fallén)

**DOI:** 10.3390/v12090908

**Published:** 2020-08-19

**Authors:** Yao Li, Ze Zhou, Mi Shen, Linquan Ge, Fang Liu

**Affiliations:** 1 College of Horticulture and Plant Protection, Yangzhou University, Yangzhou 225009, China; liyao@yzu.edu.cn (Y.L.); zz18362825460@163.com (Z.Z.); shenmi0630@126.com (M.S.); 2 Jiangsu Co-Innovation Center for Modern Production Technology of Grain Crops, Yangzhou University, Yangzhou 225009, China; 3 State Key Laboratory for Biology of Plant Diseases and Insect Pests, Institute of Plant Protection, Chinese Academy of Agricultural Sciences, Beijing 100081, China

**Keywords:** *Laodelphax striatellus*, rice stripe virus, ubiquitin-conjugating enzyme E2, viral accumulation

## Abstract

The ubiquitin–proteasome system (UPS) is an essential protagonist in host–pathogen interactions. Among the three classes of enzymes in the UPS, ubiquitin-conjugating enzyme E2 plays a dual role in viral pathogenesis; however, the role of insect E2s in interactions with plant viruses is unclear. Twenty E2-encoding genes in *Laodelphax striatellus*, the small brown planthopper, were identified and classified into 17 groups by transcriptomic and phylogenetic analysis. Full-length cDNAs of four *LstrE2s* (*LstrE2 A/E/G2/H*) were obtained by rapid-amplification of cDNA ends (RACE-PCR) analysis. Expression of the four *LstrE2s* showed tissue- and development-specific patterns. RT-qPCR analyses revealed that Rice stripe viruse (RSV) infection increased the level of *LstrE2 A/E/G2/H*. Further study indicated that repression of *LstrE2 E* via RNAi caused significant increases in the expression of RSV coat protein mRNA and protein levels. These findings suggest that LstrE2 E inhibits RSV accumulation in the planthopper body. Understanding the function of LstrE2 E in RSV accumulation may ultimately result in the development of novel antiviral strategies.

## 1. Introduction

Ubiquitin (Ub) and ubiquitin-like (Ubl) proteins are highly-conserved molecules that generally contain around 100 amino acids that can be covalently attached to protein substrates through a versatile and reversible modification known as ubiquitination [1,2,3]. Ubiquitination is a dynamic posttranslational modification that contributes to virtually all aspects of cell biology including cellular proliferation, DNA repair, apoptosis, and antigen processing [4,5,6]. Protein ubiquitination is a three-step enzymatic process that requires a series of enzymes, including ubiquitin-activating enzyme (E1), ubiquitin conjugating enzyme (E2), and ubiquitin ligase (E3). Generally, the process starts with the activation of Ub or Ubl by E1, followed by E2 and E3, which transfer and conjugate the activated Ub or Ubl to a lysine residue within the protein substrate [2]. The most common outcome of ubiquitination is protein substrate recognition and degradation by the 26S proteasome [7]. Among the ubiquitin enzymes, E2 carries the activated Ub/Ubl from the Ub/Ubl-E1 thioester to E3 or occasionally completes the conjugation of Ub/Ubl to target proteins in the absence of E3 [4,8]. During this modification, E2 determines the processivity and topology of polyubiquitin chain formation, which ultimately regulates the fate, function, and interaction of target proteins [9,10]. Most insect species contain 20 or more E2s [11], and many E2s were mistakenly considered to function as auxiliary elements in ubiquitination; consequently, E2 functionality has been under-investigated in insects. 

Given the importance of E2 enzymes in regulating the fate and function of target proteins, it is not surprising that E2 plays a vital role in the infection, replication, and movement of viruses. Accumulating evidence has demonstrated that host E2s function as a double-edged sword in viral pathogenesis and exhibit both antiviral and proviral activities [12,13]. On one hand, host E2s may function as an antiviral defense mechanism to eliminate viral components. The human E2 UBC5 (also known as UBE2D1) is required for the activation of TANK-binding kinase 1 (TBK1) through the mitochondrial antiviral signaling protein (MAVS), thereby improving antiviral immunity [14,15]. Moreover, an E2 (E2 D) from Chinese white shrimp inhibited the replication of white spot syndrome virus (WSSV) [16]. In plants, E2 (Ubc3) and RFP1 coordinately conjugate the tomato yellow leaf curl China virus (TYLCCV) βC1 protein with Ub for degradation by the 26S proteasome [17,18]. On the other hand, viruses deploy multiple strategies to manipulate E2 enzymes to favor viral infection and replication. In humans, Ubl-conjugating enzyme (SUMO-conjugating enzyme, Ubc9) sumoylates papillomavirus E1 protein, which promotes viral replication [19]. Furthermore, two tomato E2s (Cdc34p and Rad6/Ubc2) function as components of tombusvirus replication complexes that function in recruiting ESCRT (endosomal sorting complexes required for transport) proteins [20,21,22,23]. Another tomato E2 enzyme, SlUBC3, was hijacked by the βC1 protein of cotton leaf curl Multan virus (CLCuMV) to facilitate virus infection and symptom development [24]. Similar roles for the SUMO-conjugating enzyme E2 (SCE1) were found in *Nicotiana benthamiana* and *Arabidopsis thaliana* infected by geminivirus and Turnip mosaic virus, respectively [25,26,27]. These findings illustrate the diverse interaction between host E2 enzymes and viral pathogens in animals and plants; however, the role of insect E2s in viral infection remains unclear.

Rice stripe virus (RSV), a typical member of the genus *Tenuivirus*, is transmitted by *Laodelphax striatellus* (small brown planthopper) in a persistent, circulative-propagative manner; moreover, small brown planthopper has caused serious yield losses in rice production in East Asia [28,29]. The genome of RSV consists of four single-stranded, negative sense (ambisense) RNA molecules that encode the following proteins: RNA-dependent RNA polymerase (RdRP), RNA silencing suppressor (NS2), putative membrane glycoprotein (NSvc2), NS3, nucleocapsid protein (CP), nonstructural disease-specific protein (NS4), and movement protein (NSvc4) [30,31,32,33,34,35,36]. RSV particles initially establish infections in the midgut epithelium and then pass through various tissues and ultimately reach the salivary glands into healthy plants or ovaries to offspring [37]. Recently, a report demonstrated that RSV NS3 protein can hijack the 26S proteasome by interacting directly with the small brown planthopper RPN3 protein [38]. This observation implies that the ubiquitin proteasome system of small brown planthopper may function in RSV infection. However, the planthopper E2 enzymes involved in RSV infection have not been identified to date.

In this study, transcriptomic and phylogenetic analysis revealed that the twenty E2-encoding genes in small brown planthopper (*LstrE2*) could be classified into 17 groups. Four *LstrE2s* (*LstrE2 A/E/G2/H*) were sequenced and expression was analyzed in viruliferous and naïve planthopper. The results indicated that the four *LstrE2s* may have roles in mediating RSV infection; furthermore, the repression of *LstrE2 E* facilitated RSV accumulation in the planthopper body. 

## 2. Materials and Methods 

### 2.1. Plants and Insects 

Rice cv. Wuyuiing 3 was maintained in a growth incubator at 25 ± 1 °C, 80% ± 5% RH, and a 12-h light–dark photoperiod. Naïve (RSV-free small brown planthoppers) and viruliferous strains (small brown planthoppers which acquired RSV from parents) of *L. striatellus* were originally collected from Jiangsu province, China, and maintained in the laboratory for seven years. Both naïve and viruliferous *L. striatellus* were reared independently on seedlings of rice cv. Wuyujing 3 in glass beakers containing soil at a depth of 5 cm. The offspring of individual females were collected and analyzed via Dot-ELISA with a monoclonal anti-RSV CP antibody [39]. Highly viruliferous colonies were retained and used in subsequent studies. 

### 2.2. Illumina Sequencing 

Total RNA was extracted from 50 insect samples [40], and RNA concentration and purity were evaluated as described [41]. cDNA libraries were generated using established methods [40]; these were sequenced with Illumina HiSeq™ 2000 (Illumina, San Diego, CA, USA), and 125–150 bp paired-end reads were generated. Raw reads were filtered and assembled, and clean data were generated and assembled as described [40]. Small brown planthopper genome and annotation files were downloaded directly from the GigaScience repository, GigaDB (RRID: SCR004002), and clean reads were aligned with the reference genome (Bioproject number: PRJNA393384) using HISAT v. 2. (http://www.ccb.jhu.edu/software/hisat) [42]. 

### 2.3. Identification and Phylogenetic Analyses of LstrE2 Genes 

Mapped genes were manually retrieved using keywords (e.g., E2, ubiquitin, ubiquitin-conjugating enzyme, ubiquitin related enzyme, UBC, ubiquitination) and a BLASTx algorithm-based search, as described previously [40]. Predicted protein sequences of potential UBC-containing genes were recovered with ORFfinder (www.ncbi.nlm.nih.gov/orffinder/) and confirmed by BLASTp. Sequences encoding LstrE2s were initially aligned with ClustalW. Phylogenetic trees were obtained using maximum likelihood analysis in MEGA 5.2 (http://www.megasoftware.net/) and analyzed as described previously [40].

### 2.4. Cloning and Structural Analysis of LstrE2 Genes

Total RNA was isolated from 20 planthopper adults, using TRIzol reagent as recommended (Invitrogen), and RNA quality and concentration were determined by spectrophotometry (NanoDrop, Thermo Scientific, Waltham, MA, USA). RNA (500 ng) was used for reverse transcription in a 10 µl reaction volume with the PrimeScript™ RT reagent kit and gDNA Eraser as recommended (Takara, Dalian, China). Based on the mRNA sequences of *LstrE2s* obtained from transcriptomes, 5′ and 3′ rapid-amplification of cDNA ends (RACE) were conducted to obtain full-length *LstrE2* transcripts (Takara). Predicted LstrE2 proteins were subjected to Blast analysis using DNAman software (LynnonBiosoft, Los Angeles, CA, USA), and domains of predicted proteins were deduced using SMART (http://smart.embl-heidelberg.de/) [43].

### 2.5. Real-Time RT-qPCR 

To measure *LstrE2* expression and RSV copy number equivalents in small brown planthopper, total RNA was isolated from 20 intact bodies, 50 midgut/ovaries, and 100 salivary glands of adults and nymphs (female/male ratio = 1:1) using the TRIzol Total RNA Isolation Kit (Takara, Dalian, China). Total RNA concentrations were quantified, and first-strand cDNA was synthesized as described above. The primers (Table S1) used for detecting RSV copy number equivalents were designed based on *RSV CP*-specific nucleotide sequences. Similarly, *LstrE2s* and *LstrActin* (control) primers (Table S2) were designed based on *LstrE2s* and *LstrActin* sequences, respectively. RT-qPCR was conducted using a CFX96™ Real-Time PCR Detection System using reagents and parameters described previously [40]. Relative expression levels for triplicate samples were calculated using the ∆∆Ct method, and expression levels of target genes were normalized to *LstrActin*. Three technical repeats were performed for each of the three biological replicates.

### 2.6. Western Blotting

Twenty whole body samples (female/male ratio = 1:1) were collected and lysed to obtain total proteins. After adding 6× SDS loading buffer, protein samples (50 μg) were boiled for 10 min. The proteins were separated by 8–12% SDS-PAGE and transferred onto polyvinylidene fluoride (PVDF) membranes. Blots were probed with the following antibodies: anti-RSV CP (1:1000 dilution) or anti-GAPDH (1:2000 dilution). Immuno-reactive bands were detected using a goat anti-rabbit/goat anti-mouse IgG-conjugated horseradish peroxidase (HRP) antibody and a goat anti-mouse IgG-conjugated HRP antibody (Proteintech, Rosemont, IL, USA) at 1:5000 dilution. Western blots were imaged with a Chemiluminescence Detection Kit (Bio-Rad, Hercules, CA, USA) and the Molecular Imager^®^ ChemiDoc™ XRS System (Bio-Rad). 

### 2.7. RNA Interference (RNAi)

The coding sequences of *LstrE2s* and *GFP* were cloned into pMD19-T (Takara, Japan). The primers for ds*GFP* and ds*LstrE2* amplification are listed in Table S2. Using the cDNA templates obtained above, dsRNAs were synthesized using the T7 RiboMAX™ Express RNAi System kit as recommended by the manufacturer (Promega, USA). Third-instar naïve nymphs were microinjected by ds*LstrE2s* (23 nL, 2.5 μg/μL) or ds*GFP* (23 nL, 2.5 μg/μL) using an UMP3-2 UltraMicroPump (UMP3) and a SYS-Micro4 Controller (WPI, Sarasota, FL, USA) [39]. Following microinjection, nymphs were transferred and maintained on healthy rice seedlings until analyzed by immunofluorescence, RT-qPCR, or Western blot analysis. The impact of dsRNA on the expression of *LstrE2s* was evaluated by RT-qPCR.

### 2.8. Immunofluorescence Microscopy

Ten or more planthopper adults were maintained on rice plants for seven days after RNAi treatment and then dissected to obtain midgut and salivary glands. The dissected samples were fixed with 4% paraformaldehyde for 1 h. Samples were then blocked using 10% fetal bovine serum at ambient temperature for 2 h. Samples were incubated for 16 h at 4 °C with preimmune serum and anti-RSV CP antibody (1:500 dilution) before incubation with Alexa Fluor 488-labeled secondary goat anti-rabbit IgG. Salivary glands were then washed three times in: Phosphate Buffered Saline (PBS) and stained with 100 nM 2-(4-Amidinophenyl)-6-indolecarbamidine dihydrochloride (DAPI) (Sigma-Aldrich, St. Louis, MO, USA) for 2 min at room temperature. Fluorescence was observed with a Leica TCS SP8 STED confocal microscope (Leica, Wetzlar, HE, Germany). 

### 2.9. Yeast Two-Hybrid Assays 

Yeast two-hybrid assays were conducted using protocols supplied with the Yeastmaker™ Yeast Transformation System 2 (Takara-Clontech, USA). Briefly, the cDNA library of RSV was cloned as prey in plasmid vector pGADT7 using the Easy Clone cDNA library construction kit (Dualsystems Biotech); full-length *LstrE2 E* was cloned as bait in pGBKT7. Positive clones were selected on SD quadruple-dropout (QDO) medium (SD/-Ade/-His/-Leu/-Trp). To distinguish positive from false-positive interactions, we co-transformed BD-53 and AD-T, AD-LstrE2 E and BD-LstrE3, AD-LstrVg and BD-RSV CP into yeast strain Y2HGold as positive controls, respectively. ß-galactosidase activity was detected with the HTX Kit (Dualsystems Biotech).

### 2.10. GST Pull-Down Assay 

*LstrE2 E* cDNA fragments were amplified and cloned into pGEX-3X as glutathione-S-transferase (GST) translational fusions. Recombinant proteins were produced in *Escherichia coli* strain BL21 and purified. For pull-down assays, viruliferous small brown planthopper extract (1 mg), immobilized glutathione-sepharose beads (200 µL), and GST-LsTUB protein (500 µg) were added to 1 mL of pull-down buffer (50 mM Tris, 150 mM NaCl, 0.1% Triton X-100, 1 mM Phenylmethanesulfonyl fluoride (PMSF), 1% protease inhibitor cocktail [pH 8.0]) and then incubated at 4 °C for 16 h. Similarly, insect extracts were incubated with GST protein as a negative control. Beads were washed four times with pull-down buffer, and retained proteins were released by adding 2× loading buffer and incubating for 5 min at 95 °C. Proteins were then separated by SDS–PAGE and detected using anti-GST (Cusabio, China) and anti-LstrE3 antibodies (prepared in our laboratory).

## 3. Results

### 3.1. Identification and Classification of LstrE2s

**Figure 1:**
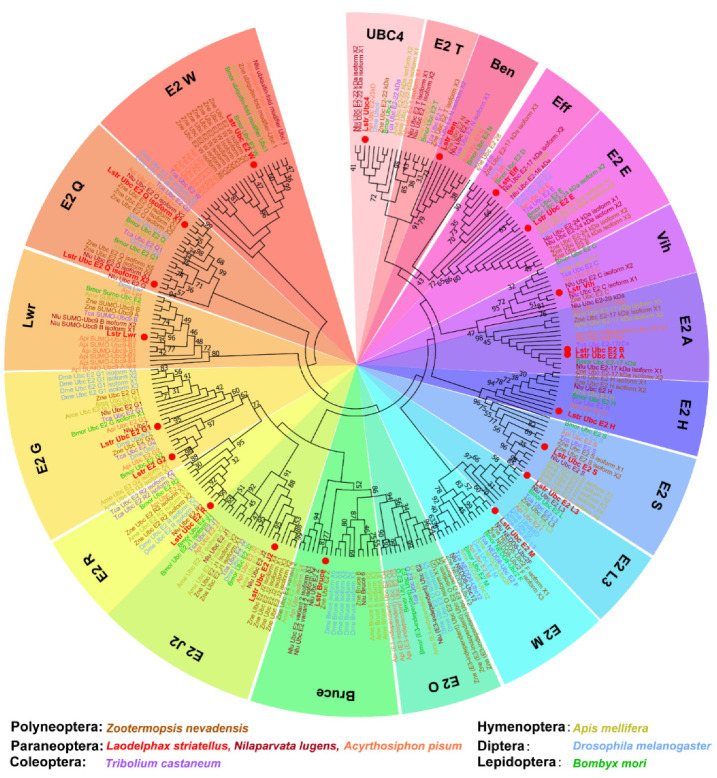
**Phylogenetic analysis of ubiquitin-conjugating enzyme E2s in different insect orders.** Amino acid sequences were aligned using ClustalW (www.ebi.ac.uk/clustalW), and a maximum likelihood tree was generated using MEGA 7.0. Nodes with distance bootstrap values (100 replicates) are shown. Different species are marked in different colors. Red dots indicate E2s in *L. striatellus*. Abbreviations: Zne, *Zootermopsis nevadensis*; Lstr, *Laodelphax striatellus*; Nlu, *Nilaparvata lugens*; Api, *Acyrthosiphon pisum*; Tca, *Tribolium castaneum*; Ame, *Apis melifera*; Dme, *Drosophila melanogaster*; Bmo, *Bombyx mori*.

The transcriptomes of intact small brown planthopper bodies were sequenced using the Illumina HiSeq™ 2000 platform. More than 42.94 million clean reads were obtained from each transcriptome and over 60.9% mapped to the small brown planthopper genome (Table S2). The mapped genes annotated as candidates of ubiquitin-conjugating enzyme (E2) were screened with a BLASTx algorithm-based search using known E2 genes form *Drosophila melanogaster* and other insect species. Twenty E2 genes were identified in small brown planthopper, which included the ubiquitin-conjugating and ubiquitin-like conjugating enzymes (Table 1). A phylogenetic tree was constructed for LstrE2s and proteins containing ubiquitin-conjugating (UBC) domains from six insect species in orders Polyneoptera, Paraneoptera, Coleoptera, Hymenoptera, Diptera, and Lepidoptera (Figure 1). Almost all insect E2s were grouped into 19 clades comprised of E2 W, E2 Q, lessright (Lwr), E2 G, E2 R, E2 J2, BIR-repeat containing ubiquitin-conjugating enzyme (Bruce), E2 O, E2 M, E2 L3, E2 S, E2 H, E2 A, vihar (Vih), E2 E, effete (Eff), bendless (Ben), E2 T, and UBC4; this assignment is consistent with the nomenclature of E2 in human cells [44]. The 20 LstrE2s were distributed among these 17 groups, which suggested shared functions with other insect E2s.

### 3.2. Cloning and Sequence Analysis of Four LstrE2s 

**Figure 2:**
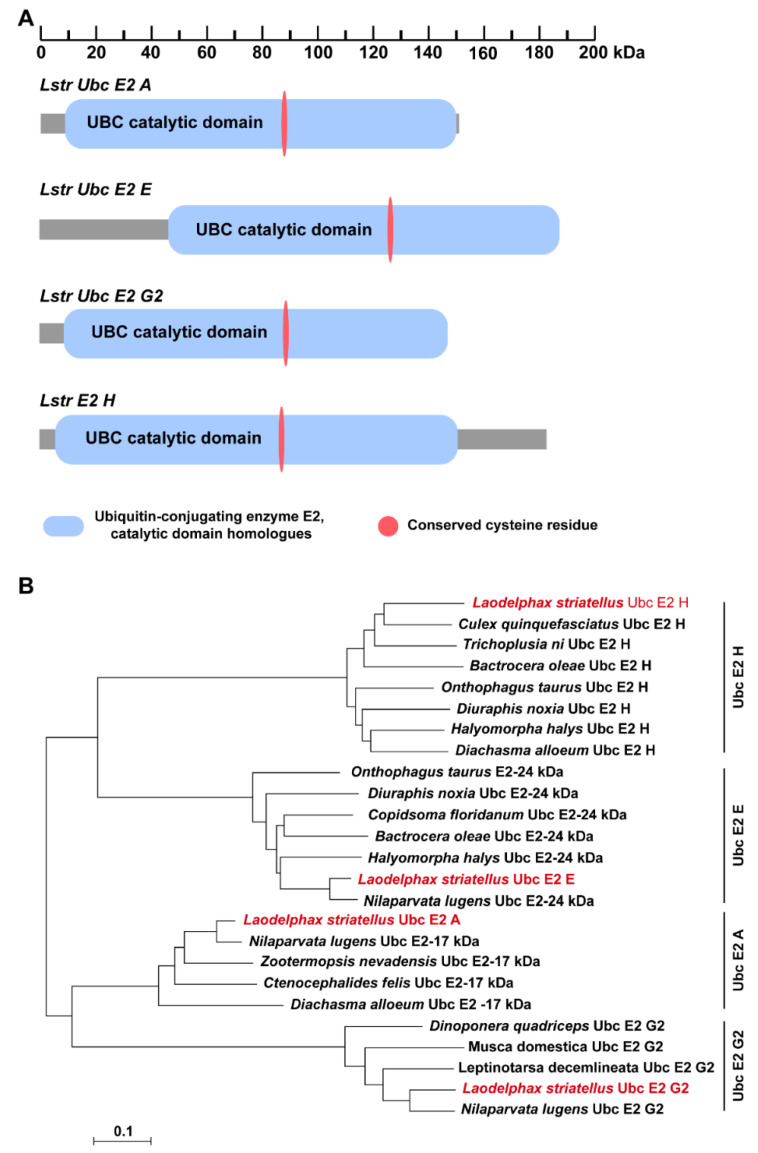
**Conserved motifs and phylogenetic analysis of selected E2 enzymes.** (**A**) The ubiquitin conjugating enzyme (UBC) catalytic domain and conserved cysteine residue in LstrE2 A/E/G2/H are indicated by blue and red ovals, respectively. (**B**) Phylogenetic analysis of E2s from selected fifteen insect species including *Laodelphax striatellus*, *Nilaparvata lugens*, *Culex quinquefasciatus*, *Trichoplusia ni*, *Bactrocera oleae*, *Onthophagus taurus*, *Diuraphis noxia*, *Halyomorpha halys*, *Diachasma alloeum*, *Copidsoma floridanum*, *Zootermopsis nevadensis*, *Ctenocephalides felis*, *Dinoponera quadriceps*, *Musca domestica*, *Leptinotarsa decemlineata*. Amino acid sequences were aligned using ClustalW, and a distance neighbor-joining tree was generated using MEGA 7.0. Nodes with distance bootstrap values (1000 replicates) are shown. Red font indicates the four E2s from *L. striatellus*.

Full-length cDNA sequences of four *LstrE2s* were cloned from planthopper adults using conserved *LstrE2* sequences as an in-silico probe. *LstrE2 A* (GenBank accession no. MT334578) is a 1089-bp cDNA that encodes a putative protein of 151 amino acids with a theoretical molecular weight (MW) and isoelectric point (pI) of 17,200.44 and 5.69, respectively. *LstrE2 E* (GenBank accession no. MT334581) is a 1209-bp full-length cDNA encoding a putative protein of 188 amino acids (MW, 20,477.39; pI, 8.79). *LstrE2 G2* (GenBank accession no. MT334580) is an 854-bp cDNA that encodes a putative protein of 144 amino acids (MW, 15,853.06; pI, 4.39). *LstrE2 H* (GenBank accession no. MT334579) is a 1303-bp full-length cDNA encoding a putative protein of 184 amino acids (MW: 20,911.44, pI: 4.89). SMART analysis showed that each *LstrE2* contained one UBC catalytic domain and a conserved cysteine residue (Figure 2A). Phylogenetic analysis revealed that the four LstrE2s had high sequence identity with other insect E2s deposited in the NCBI database and were very closely related to E2s in *Nilaparvata lugens* (Figure 2B). 

### 3.3. Expression Analysis of the Four LstrE2s

**Figure 3:**
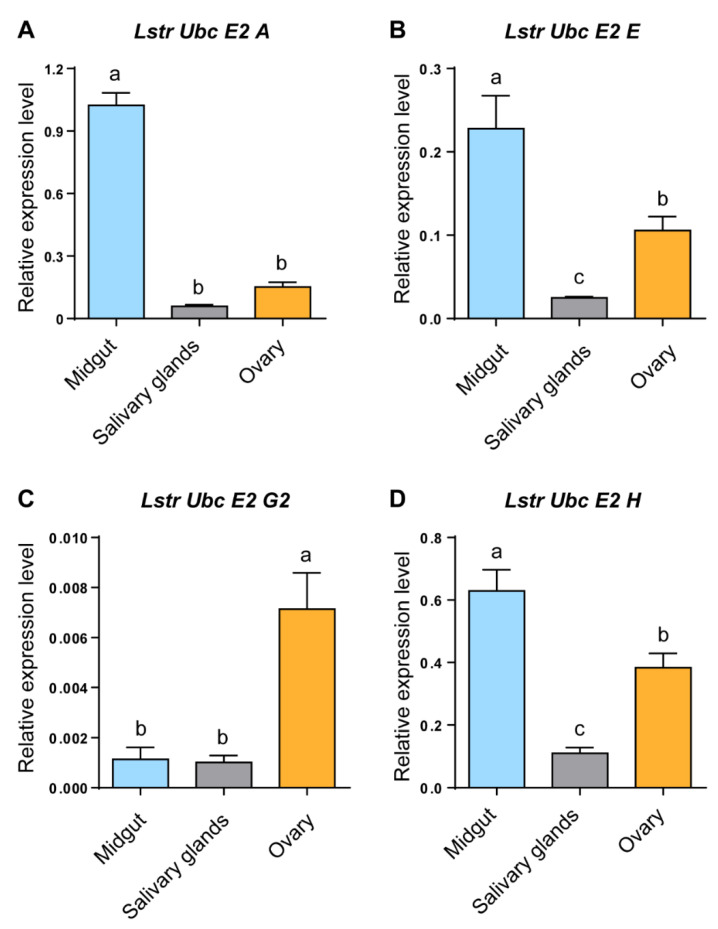
**The expression of *LstrE2 A/E/G2/H* in different tissues of small brown planthopper adults.** RT-qPCR analysis of (**A**) LstrE2 A, (**B**) LstrE2 E, (**C**) LstrE2 G2, and (**D**) LstrE2 H expression in midguts, salivary glands, and ovaries of adults. In total, 50 midguts, 100 salivary glands, and 50 ovaries were considered to be a single replicate, and each treatment contained three replicates. All expressions are relative to first column in panel A. Bars labeled with different letters indicate significant differences in expression levels using RT-qPCR (*p* < 0.05).

**Figure 4:**
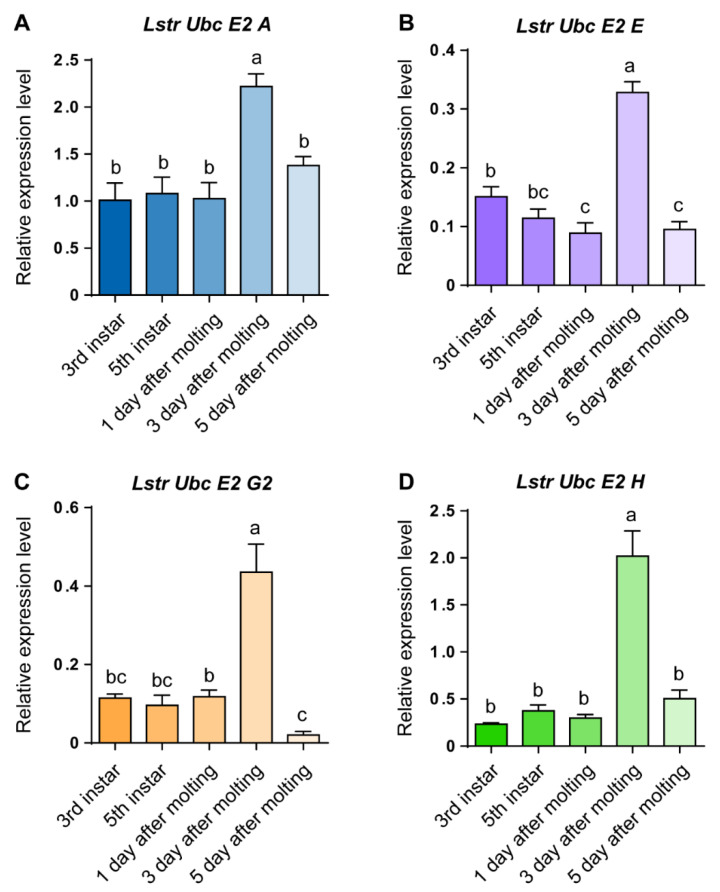
**The expression of *LstrE2 A/E/G2/H* at different developmental stages of small brown planthopper.** RT-qPCR analysis of (**A**) LstrE2 A, (**B**) LstrE2 E, (**C**) LstrE2 G2, and (**D**) LstrE2 H expression in 20 insects at 3rd instar, 5th instar, 1 day after molting, 3 days after molting, and 5 days after molting. In total, 20 insects were considered to be a single replicate, and each treatment contained three replicates. All expressions are relative to first column in the panel A. Bars labeled with different letters indicate significant differences in expression (*p* < 0.05).

RT-qPCR was used to evaluate *LstrE2 A/E/G2/H* mRNA expression in different tissues and developmental stages of small brown planthopper. Three *LstrE2s* (*LstrE2 A*, *E*, *H*) were more highly expressed in midgut than in salivary glands or ovaries (Figure 3A,B,D), while the *LstrE2 G2* was most highly expressed in ovaries (Figure 3C). The four *LstrE2* expression profiles in the five developmental stages were very similar. The highest transcription level was detected in planthopper adults sampled three days after molting (Figure 4). 

### 3.4. Rice Stripe Virus Increases LstrE2s Expression in Small Brown Planthopper Adults

**Figure 5:**
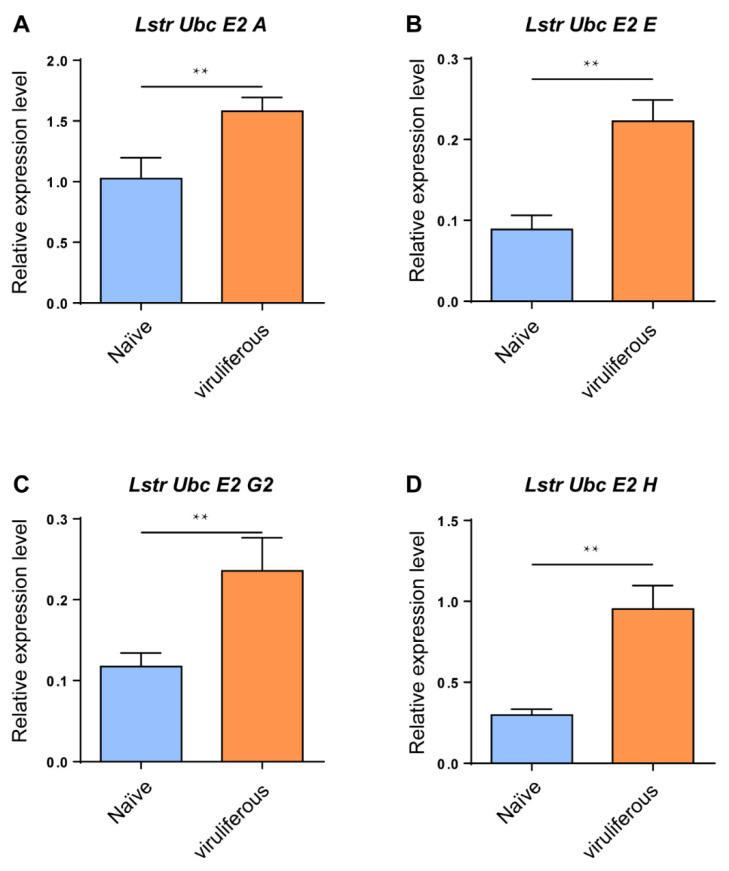
***LstrE2 A/E/G2/H* expression in virus-naïve and viruliferous small brown planthopper.** RT-qPCR analysis of (**A**) *LstrE2 A*, (**B**) *LstrE2 E*, (**C**) *LstrE2 G2*, and (**D**) *LstrE2 H* expression in naïve and viruliferous adults. In total, 20 insects were considered to be a single replicate, and each treatment contained three replicates. All expressions are relative to first column in the panel A. Means ± S.E; *t*-test analysis, ** *p* < 0.01.

RT-qPCR analyses were conducted to evaluate expression levels of the four *LstrE2s* in viruliferous and virus-naïve planthopper adults. The mRNA expression levels of the four *LstrE2s* were significantly upregulated in viruliferous planthopper adults (Figure 5); for example, the expression of *LstrE2 A*, *LstrE2 E*, *LstrE2 G2*, and *LstrE2 H* increased by 54.2%, 220.7%, 100.7%, and 150.5%, respectively, when compared to viruliferous-naïve planthopper adults (Figure 5A–D). 

### 3.5. Repression of LstrE2 E via RNAi Increases RSV Load in Small Brown Planthopper

**Figure 6:**
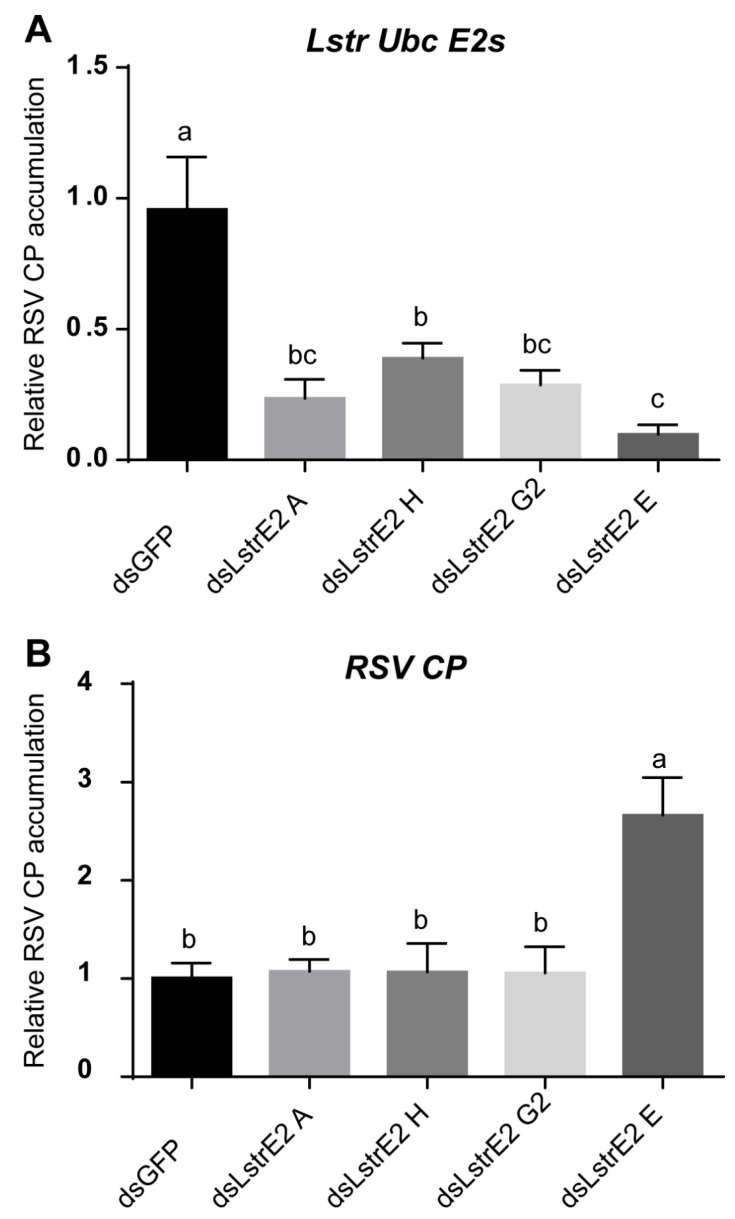
**The effect of *LstrE2 A/E/G2/H* knockdown on Rice stripe viruse load in small brown planthopper.** RT-qPCR analysis of (**A**) *LstrE2* expression and (**B**) RSV coat protein (CP) accumulation in dsGFP-, ds*LstrE2 A*-, ds*LstrE2 E*-, ds*LstrE2 G2*-, and *dsLstrE2 H*-treated planthopper. In total, 20 treated nymphs were considered to be a single replicate, and each treatment contained three replicates. Means ± S.E. One-way ANOVO analysis: different letters above bars (a,b,c) indicate significant differences of expression level between treatments.

**Figure 7:**
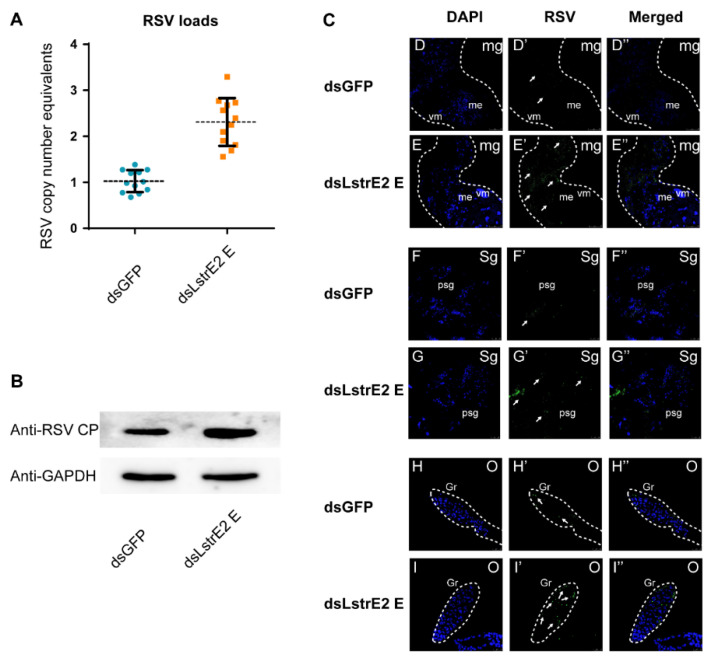
**The effects of *LstrE2 E* knockdown on RSV accumulation and CP levels in viruliferous small brown planthopper.** (**A**) RT-PCR analysis of RSV copy number equivalents in single ds*LstrE2 E*- and ds*GFP*-treated planthopper. Single insect was considered to be a single replicate, and each treatment was replicated twelve times; means ± S.E. (**B**) Western blot analysis of RSV CP in ds*LstrE2 E*- and ds*GFP*-treated adults. In total, 20 treated adults were mixed and used for protein extraction. Glyceraldehyde-3-phosphate dehydrogenase (GAPDH) gene was used as control. (**C**) Immunofluorescence in midgut, salivary glands and ovaries of viruliferous planthopper treated with ds*LstrE2 E* and ds*GFP*. Anti-RSV CP (Alexa Fluor 488, green) and DAPI (blue) were used as fluorescent probes. Each treatment was replicated five times. Abbreviations: mg, midgut; sg, salivary glands; o, ovary; vm, visceral muscle; me, midgut epithelium; psg, principal salivary glands; gr, germarium. Bar = 50 µm.

To further explore the function of the four LstrE2s in RSV infection, 3rd instar viruliferous planthopper nymphs were microinjected with 0.5 mg/mL dsRNAs derived from *GFP* (*dsGFP*) or *LstrE2 A/E/G2/H* (ds*LstrE2 A/E/G2/H*). At seven days post-dsRNA treatment, RT-qPCR analyses showed that *LstrE2* mRNA expression levels in the corresponding dsLstrE2-treated planthoppers were significantly reduced by 61.4–90.4% compared to controls (*dsGFP*-treated planthoppers) (Figure 6A). These results indicated that RNAi-mediated knockdown of the four *LstrE2s* was highly effective. RT-qPCR indicated that only the ds*LstrE2 E* treatment caused an increase in RSV copy number equivalents of viruliferous planthopper (Figure 6B). Furthermore, RSV load and distribution were examined in planthopper whole bodies and different tissues via RT-qPCR, Western blotting, and confocal microscopy. RSV copy number equivalents were elevated in the 12 ds*LstrE2 E*-treated planthoppers (Figure 7A), which agrees with results obtained with mixed samples (Figure 6B). Immunoblotting indicated that the trend of RSV CP protein production was consistent with changes in mRNA expression (Figure 7B). Confocal microscopy indicated that RSV particles were also present in midgut, salivary glands, and ovaries of ds*LstrE2 E*-treated planthoppers; furthermore, RSV immunofluorescence was more intense in ds*LstrE2 E*-treated than ds*GFP*-treated planthopper (Figure 7C, Figure S1). These results indicated that repression of *LstrE2 E* facilitated RSV accumulation in the planthopper body.

### 3.6. LstrE2 E Does Not Directly Interact with RSV Proteins

**Figure 8:**
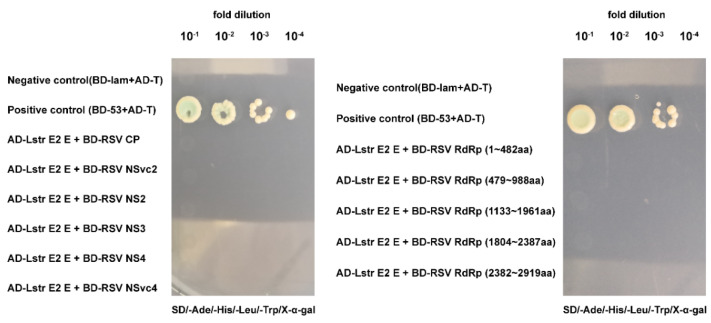
**Interactions between LstrE2 E and RSV proteins using yeast two-hybrid (Y2H) assays.** Yeast cells were diluted 10^−1^ to 10^−4^ and plated onto quadruple-dropout (QDO) (SD-trp-leu-his-ade-20 mM3-AT) medium. Colonies growing on QDO were also assayed for β-galactosidase activity (blue coloration). Positive control, AD-T + BD-53; negative control, AD-T + BD-lam. Abbreviations: AD, activation domain cloned in pGADT7; BD, bait domain in pGBKT7.

Considering that LstrE2 E may mediate RSV load by binding viral proteins, we used yeast two-hybrid assays to evaluate whether LstrE2 E interacts with seven known RSV proteins (CP, SP, NS2, NS3, NSvc2, NSvc4, and RdRp). Full-length LstrE2 E was used as bait, and each of the six intact proteins (CP, SP, NS2, NS3, NSvc2, and NSvc4) and five truncated RdRp mutants were used as prey. All yeast strains failed to grow on synthetic dextrose dropout medium (Figure 8, Figure S2). This result suggested that LstrE2 E does not directly interact with RSV proteins.

## 4. Discussion

Phylogenetic analyses showed that 263 E2 enzymes from eight insect species could be categorized into 19 groups (Figure 1). There are few studies of insect E2s; consequently, the nomenclature and classification of E2s in insects is chaotic. Thus, we adopted nomenclature for small brown planthopper UBC-domain-containing proteins based on their relatedness to *Drosophila* and human orthologues [44]. LstrE2 E and LstrEff occur in higher eukaryotes as UBE2D1–4 and UBE2E1–3, which are involved in the degradation of short-lived and abnormally folded proteins [45]. The LstrE2 A/LstrE2 B and LstrE2 G1/LstrE2 G2 proteins also appear pairwise in humans [46]. *LstrBruce*, which encodes giant E2 protein, is present in both *Drosophila* and humans [47], whereas E2 O and E2 T have not been identified in small brown planthopper. Insect E2s in the same clade share a similar structure, which suggests that they may have related functions and possibly target similar lysine residues to construct polyubiquitin chains.

The UBC E2 family has expanded and diversified during evolution, and many E2s exist in both prokaryotes and eukaryotes. The E2 family includes both proteins and inactive variants that range in number from up to 20 in prokaryotes and over 40 in multicellular eukaryotes. For example, 12 and 16 E2s were identified in the algae *Ostreococcus tauri* and yeast *Saccharomyces cerevisiae*, respectively [44,48]; in plants, 37, 48, and 75 E2s were identified in *Arabidopsis thaliana*, rice, and maize, respectively [47,48,49]. Twenty and 37 E2s were identified in *Caenorhabditis elegans* and humans [11,46]. In this study, 20 E2s were initially identified in planthopper via transcriptome analysis; 18 were ubiquitin-conjugating enzymes and two were Ubl-conjugating enzymes (SUMO- and NEDD8-conjugating enzymes) (Figure 1). The number of E2s in planthopper is smaller than the numbers in plants and humans, which is likely due to developmental complexity.

Expression profile analysis revealed that the four *LstrE2s* are present in all planthopper tissues and exhibit development- and tissue-specific expression patterns (Figure 3 and Figure 4). The expression of the four *LstrE2s* peaked at three days after molting in planthopper adults, suggesting that they function in ubiquitination at this developmental stage. *LstrE2 A/E/H* were primarily expressed in planthopper midgut and were highly expressed during RSV infection; these results suggest a role for LstrE2 A/E/H in RSV infection in the midgut. Based on the high expression of *LstrE2 G2* in ovaries and after RSV infection, we speculate that LstrE2 G2 may be involved in transovarial transmission of RSV. These results demonstrate that the four LstrE2s play important roles in the planthopper immune response, which warrants further investigation.

We show that *LstrE2 E* was highly expressed in response to RSV infection, and repression of *LstrE2 E* facilitated RSV accumulation in the planthopper body. These findings indicated that the role of LstrE2 E in RSV accumulation is consistent with the role of animal and plant E2s in host defense mechanism against virus, as described above. However, Y2H analysis showed that LstrE2 E did not directly interact with RSV proteins. LstrE2 E inhibited RSV accumulation through an unknown antiviral defense mechanism. The human E2 enzyme UbcH7 functioned with the E3 SCF complex to ubiquitinate and degrade papillomavirus E7 protein [50], and the plant E2 Ubc3 and E3 Ligase RFP1 coordinately provide an antiviral mechanism that targeted a viral protein for degradation [17,18]. Based on these reports, it is plausible that LstrE2 E may also function with E3 to degrade viral proteins, thereby inhibiting viral accumulation. Another possibility is that LstrE2 E may impact viral load by an unknown pathway. The precise mechanism of RSV inhibition by LstrE2 E warrants further investigation.

In this study, 20 E2s were identified in small brown planthopper, and full-length cDNAs were obtained for four *LstrE2s* (*LstrE2 A/E/G2/H*). Expression of the four *LstrE2s* was highest in the midgut and ovaries of planthopper adults. LstrE2 E was highly expressed during RSV infection and inhibited viral accumulation in small brown planthopper. These results suggested that *LstrE2 E* expression correlated with RSV accumulation in small brown planthopper. These results provide insights for understanding the interaction between RSV and small brown planthopper and provide new avenues to control plant disease.

## Figures and Tables

**Table 1:** Ubiquitin-conjugating and ubiquitin-like conjugating enzymes identified in small brown planthopper.

Gene Names	Reference Number	BLASTx Best Hit from *Drosophila*	Accession Number of Drosophila	Pathway Name
LstrE2 A	gene-LSTR_LSTR002412	Ubc6	Dmel_CG2013	Ubiquitin-mediated proteolysis
LstrE2 J2	gene-LSTR_LSTR004167	Ubc10	Dmel_CG5788	Ubiquitin-mediated proteolysis
LstrE2 E	gene-LSTR_LSTR008905	Ubc2	Dmel_CG6720	Ubiquitin-mediated proteolysis
LstrE2 H	gene-LSTR_LSTR010396	UbcE2H	Dmel_CG2257	Ubiquitin-mediated proteolysis
LstrE2 G2	gene-LSTR_LSTR011058	Ubc7	Dmel_CG4443	Ubiquitin-mediated proteolysis
LstrE2 M	gene-LSTR_LSTR015577	UbcE2M; ubiquitin-conjugating enzyme E2M, isoform B	Dmel_CG7375	Ubiquitin-mediated proteolysis
LstrUbc4	gene-LSTR_LSTR017241	Ubc4	Dmel_CG8284	Ubiquitin-mediated proteolysis
LstrEff	gene-LSTR_LSTR000118	eff; effete, isoform B	Dmel_CG7425	Ubiquitin-mediated proteolysis
LstrE2 R	gene-LSTR_LSTR000713	uncharacterized protein, isoform F	Dmel_CG7656	Ubiquitin-mediated proteolysis
LstrE2 W	gene-LSTR_LSTR001122	uncharacterized protein, isoform E	Dmel_CG7220	Ubiquitin-mediated proteolysis
LstrVih	gene-LSTR_LSTR002766	vih; vihar	Dmel_CG10682	Ubiquitin-mediated proteolysis
LstrBen	gene-LSTR_LSTR003814	ben; bendless, isoform B	Dmel_CG18319	Ubiquitin-mediated proteolysis
LstrLwr	gene-LSTR_LSTR005749	lwr; lesswright, isoform C	Dmel_CG3018	Ubiquitin-mediated proteolysis
LstrE2 L3	gene-LSTR_LSTR006367	uncharacterized protein, isoform B	Dmel_CG5823	Ubiquitin-mediated proteolysis
LstrE2 S	gene-LSTR_LSTR007167	uncharacterized protein, isoform B	Dmel_CG8188	Ubiquitin-mediated proteolysis
LstrE2 G1	gene-LSTR_LSTR008154	uncharacterized protein, isoform A	Dmel_CG40045	Ubiquitin-mediated proteolysis
LstrBruce	gene-LSTR_LSTR014931	Bruce; BIR repeat containing ubiquitin-conjugating enzyme, isoform B	Dmel_CG6303	Ubiquitin-mediated proteolysis
LstrE2 B	gene-LSTR_LSTR016706	uncharacterized protein, isoform C	Dmel_CG10254	Ubiquitin-mediated proteolysis
LstrE2 Q isoform X1	novel.10	UBE2Q	Dmel_CG2924	Ubiquitin-mediated proteolysis
LstrE2 Q isoform X2	novel.1252	UBE2Q	Dmel_CG4502	Ubiquitin-mediated proteolysis

## Supplementary Materials

The following are available online at https://www.mdpi.com/1999-4915/12/9/908/s1, Table S1: The primers used in this study, Table S2: Reads mapping to the reference genome.
