# Supplementary material for: Ubiquitin-Conjugating Enzyme E2 E Inhibits the Accumulation of Rice Stripe Virus in Laodelphax striatellus (Fallén)
Source: Viruses. 2020 Aug 19;12(9):908. doi: 10.3390/v12090908 (PMC7551955; doi:10.3390/v12090908)
Supplement: Supplementary file 1 [file viruses-12-00908-s001.pdf]

Table S1. The primers used in this study

Gene name	primers	Sequences
RSV CP	q-RSV-F	TGAAAGTGGCGGCTGGAA
	q-RSV-R	CCACCGAGGACACTATCCCATA
LsActin	Actin-F	GTCTCACACACAGTCCCCATCTATG
	Actin-R	TCGGTCAAGTCACGACCAGC
GFP	GFP-F	AAGGGCGAGGAGCTGTTCACCG
	GFP-R	CAGCAGGACCATGTGATCGCGC
LstrE2 A	Lstr E2 A-F	ATAGGGTGTTGAACTGCT
	Lstr E2 A-R	AGATTAGGGTCTAATGGC
	q-Lstr E2 A-F	TATCAGCCATTCTTACTTCT
	q-Lstr E2 A-R	TCTCGTATTCCCGTCTAT
LstrE2 H	Lstr E2 H-F	TTAGTAGCCGTGGACATC
	Lstr E2 H-R	TTTTGGGAACAATAGGTG
	q-Lstr E2 H-F	ATTATCCCTTCAAATCGC
	q-Lstr E2 H-R	CAGACAAACAGTGCCAGA
LstrE2 G2	Lstr E2 G2-F	GAGTGGAATTTTAGATTTG
	Lstr E2 G2-R	ATACAGGCTCCTAGAACG
	q-Lstr E2 G2-F	TGCCAGTCCTATCAACGA
	q-Lstr E2 G2-R	GGGAATACACCACCCTCA
LstrE2 E	Lstr E2 E-F	GCAGTGGTATCAACGCAGAG
	Lstr E2 E-R	ACGATTGTCAAGCCGAAC
	q-Lstr E2 E-F	TCAGTCTACGAAGGTGGCG
	q-Lstr E2 E-R	CCTGGCTATTGATGTTGC

Table S2. Reads mapping to the reference genome

Sample	Total_reads	Total_map	Unique_map	Multi_map	Positive_map	Negative_map	Proper_map
SBPH whole body 1	49312682	30031982(60.9%)	27972416(56.72%)	2059566(4.18%)	13965239(28.32%)	14007177(28.4%)	24361722(49.4%)
SBPH whole body 2	60196634	37248814(61.88%)	35101689(58.31%)	2147125(3.57%)	17521733(29.11%)	17579956(29.2%)	30447268(50.58%)
SBPH whole body 3	55400060	36062951(65.1%)	33933296(61.25%)	2129655(3.84%)	16923889(30.55%)	17009407(30.7%)	29516912(53.28%)

Figure S1

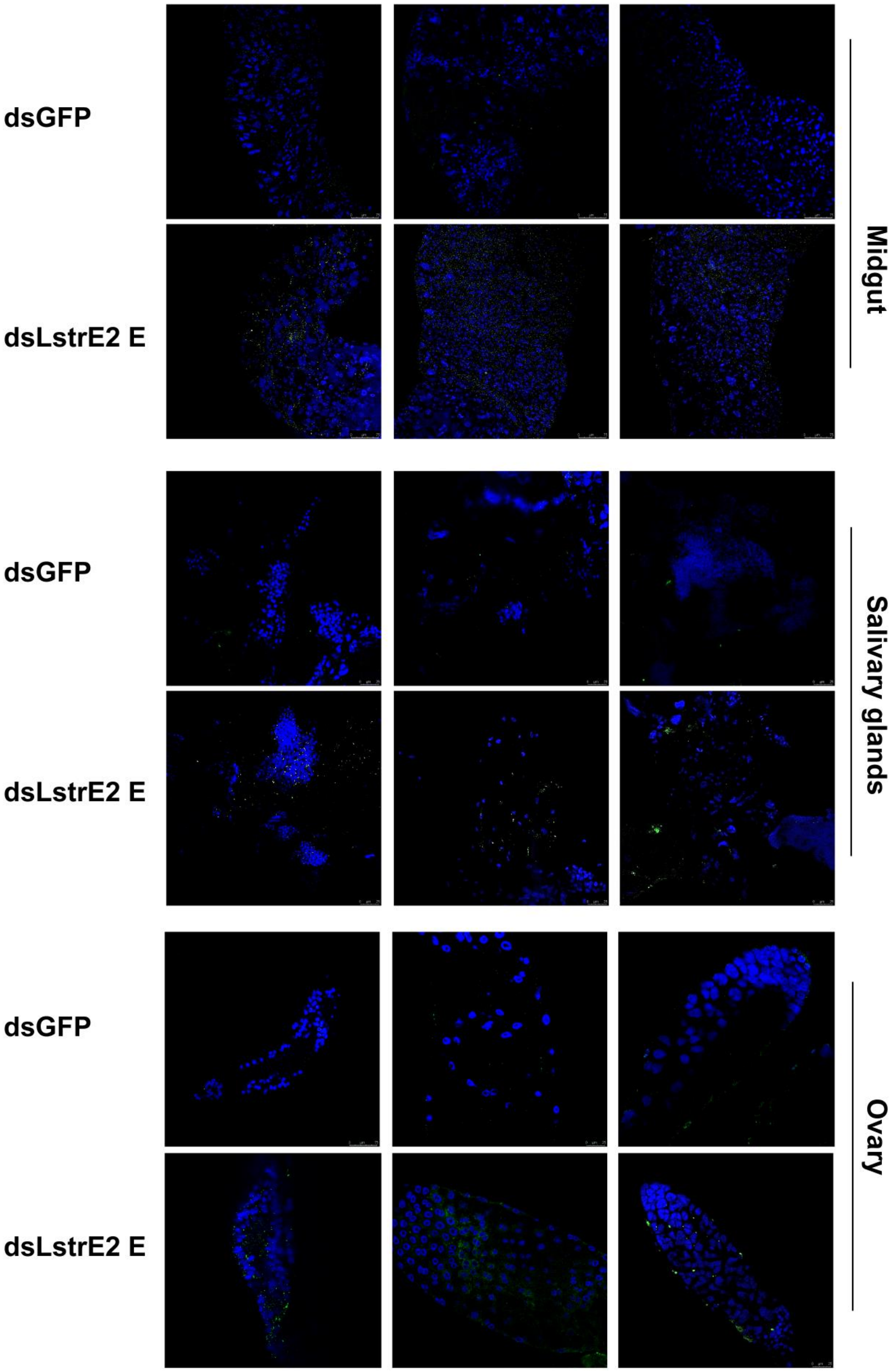
dsGFP
dsLstrE2 E
dsGFP
dsLstrE2 E
dsGFP
dsLstrE2 E
Midgut
Salivary glands
Ovary

Figure S2

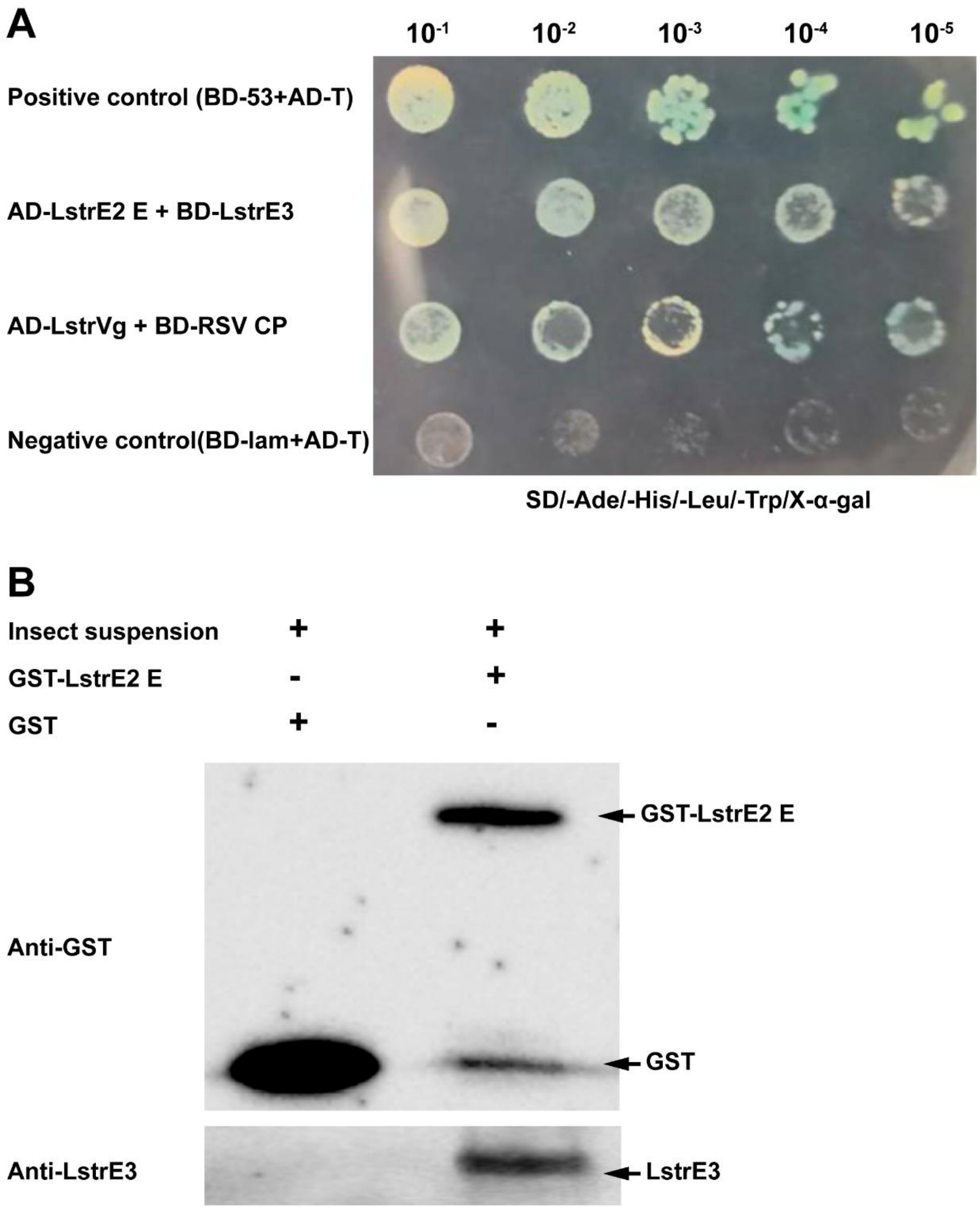
A
10^{-1}
10^{-2}
10^{-3}
10^{-4}
10^{-5}
Positive control (BD-53+AD-T)
AD-LstrE2 E + BD-LstrE3
AD-LstrVg + BD-RSV CP
Negative control(BD-lam+AD-T)
SD/-Ade/-His/-Leu/-Trp/X-α-gal
B
Insect suspension + +
GST-LstrE2 E - +
GST + -
Anti-GST
GST-LstrE2 E
GST
Anti-LstrE3
LstrE3
